# Editorial: Fraud and Corruption in Healthcare

**DOI:** 10.3389/fpubh.2022.921254

**Published:** 2022-06-01

**Authors:** Yuriy Timofeyev, Mihajlo Jakovljevic

**Affiliations:** ^1^Graduate School of Business, HSE University, Moscow, Russia; ^2^Institute of Advanced Manufacturing Technologies, Peter the Great St. Petersburg Polytechnic University, St. Petersburg, Russia; ^3^Institute of Comparative Economic Studies, Hosei University, Tokyo, Japan; ^4^Department of Global Health Economics and Policy, University of Kragujevac, Kragujevac, Serbia

**Keywords:** fraud, corruption, abuse, healthcare, prevention, medical doctors

Healthcare is usually associated with essential positive externalities toward human beings. Many nations aim to achieve not only universal health coverage, but also improve the quality of healthcare outcomes. Both goals require relatively large expenditure (1). Although healthcare efficiency ratios vary among countries (2), corruption and fraud remain among the major factors of government inefficiencies at macro levels and of healthcare outcomes at micro levels. Corruption fundamentally weakens health systems and disrupts progress toward the goal of universal health coverage (3). Importantly, in contrast to unintentional medical errors, fraud and corruption refer to intentional violations of “good” practices by doctors, administrators, etc. (4).

The scale of losses due to fraud and corruption in healthcare is impressive as it accounts for approximately $455 billion of the $7.35 trillion spent on healthcare annually worldwide (National Academies of Sciences, Engineering, and Medicine, 2018). Sometimes one can observe outstanding examples of either (groups of) perpetrators or victims [e.g., (5)], especially when considering organized crime like the so-called ”Mirzoyan-Terdjanian organization” (6).

The forms of fraud and corruption in healthcare are similar to those in other industries. However, there are several specific ones, such as “upcoding” marginal patients to having had a related, but more complex illness or treatment in order to obtain higher reimbursement (7) or pharmaceutical fraud, which includes misbranding, counterfeiting, promoting the off-label use of drugs, etc. (8). Sommersguter-Reichmann et al. (9) provide an overview of, and links among, various corruption typologies in European and US healthcare. Remarkably, fraudsters very quickly adapt to the changing situation and either invent new forms or modify the existing forms of fraud. In 2021, for example, the US pharmaceutical company Pfizer announced it had identified counterfeit versions of its coronavirus vaccine in Mexico and Poland (10).

Notably, evidence on fraud and corruption in healthcare is limited in many countries for formal or informal reasons. The five articles included in this Research Topic provide novel insights from four different countries (South Africa, Nigeria, South Korea, and the US) on issues related to the nature, scope, and consequences of law and/or ethics violation in healthcare.

Mantzaris and Pillay, in their article “*Legal Profession and Corruption in Health Care: Some Reflective Realities in South Africa*,” analyze and dissect the involvement of legal practitioners in illegal and/or fraudulent acts, especially in litigation associated with issues of medical negligence. The authors reasonably conclude that the enhancement of effectiveness, professionalism, and efficiency have little or no impact, unless they are founded on moral norms.

Obodoechi et al., in their article “*Health Worker Absenteeism in Selected Health Facilities in Enugu State: Do Internal and External Supervision Matter?*” examine the role of internal and external supervision arrangements on health worker absenteeism in Nigeria. The authors conclude that the existing system of external supervision of absenteeism does not work. They consider a more holistic approach to the experiences of health workers as a potential solution.

Dongkyu, in his paper “*The Anticipation of Crime and Corruption Problems Due to Expansion of Telemedicine: A Study Based on Korea Medical Crime Investigation System*,” investigates aspects of fraud driven by the rapid expansion of telemedicine, such as patients' illegal use of unlicensed devices, data abuse, or drug abuse. The author suggests that it is necessary to make internationally standard institutional regulations.

O'Mathúna, in his article “*Ivermectin and the Integrity of Healthcare Evidence During COVID-19*,' explores the ethics of how healthcare evidence must be critically appraised, especially during a pandemic. The author argues that evidence and ethics must be combined to ensure that the patients' needs and safety are given top priority in the production and further application of academic publications.

Jung and Kim, in their article “*Insurance Fraud in Korea, Its Seriousness, and Policy Implications*,” draw attention to the problem of massive victimization of insurance companies, which continues to be associated with a growing social burden. Although this article is geographically tailored to Korea, the recommendations provided are relevant to different extents in many other countries.

Summing up, although fraud and corruption cannot be eradicated completely, there are many effective preventive mechanisms, which can help to improve healthcare system outcomes and, literally, save human lives.

## Author Contributions

YT prepared the manuscript draft, while MJ revised it for important intellectual content. All authors contributed to the article and approved the submitted version.

## Conflict of Interest

The authors declare that the research was conducted in the absence of any commercial or financial relationships that could be construed as a potential conflict of interest.

## Publisher's Note

All claims expressed in this article are solely those of the authors and do not necessarily represent those of their affiliated organizations, or those of the publisher, the editors and the reviewers. Any product that may be evaluated in this article, or claim that may be made by its manufacturer, is not guaranteed or endorsed by the publisher.
